# COVID-19 and global equity for health: The good, the bad, and the wicked

**DOI:** 10.1371/journal.pmed.1003797

**Published:** 2021-10-01

**Authors:** Elvin H. Geng, Michael J. A. Reid, Eric Goosby, Quarraisha Abdool-Karim

**Affiliations:** 1 Division of Infectious Diseases, Department of Medicine and Center for Dissemination and Implementation in the Institute for Public Health, Washington University in St. Louis, United States of America; 2 Institute for Global Health Sciences, University of California, San Francisco, California, United States of America; 3 Department of Medicine, University of California, San Francisco, California, United States of America; 4 Centre for the AIDS Programme of Research in South Africa, University of KwaZulu-Natal, Durban, South Africa; 5 Department of Epidemiology, Mailman School of Public Health, Columbia University, New York, New York, United States of America

## Abstract

Elvin Geng and co-authors discuss monitoring and achieving equity in provision of vaccines for COVID-19.

Many problems underscored by the Coronavirus Disease 2019 (COVID-19) pandemic, and highlighted in other papers in this collection [1], center on addressing the grossly unequal availability of vaccine around the world. The problem of equity in the public health response fits Rittel and Webber’s conceptualization of “wicked problems” [2] that describe complex issues that defy conventional scientific analyses. Wicked problems have many characteristics, but three stand out when contemplating an equitable response to the COVID-19 pandemic. First, wicked problems have no definitive problem statement because understanding the problem is the crux of the problem itself. Is an equitable response to COVID-19 the problem, or is inequity in vaccines a symptom of larger inequity in health, economic, and societal systems? And is the problem equity in distribution, or is the root problem scarcity due to unfair trade arrangements? Second, in wicked problems, the elusive problem formulation precludes a shared understanding of success, or even progress, by stakeholders. What would constitute an acceptably equitable response? How would we measure progress without a unified direction? Third, in wicked problems, the absence of a shared agenda (and measures of progress) undermines aligned and effective action. Without true consensus that a fair allocation of vaccines across countries is based on population size instead of disease burden (as proposed by COVAX), targets based on progress toward such a distribution would lack real commitment from global stakeholders. The answers are as critical as they are elusive, not only for how the COVID-19 pandemic evolves, but also for whether we learn from it to better navigate future threats.

COVID-19 is not the first wicked problem evident in global public health, however, and the recent past may offer some lessons. Three decades ago, HIV presented an urgent, complex, dynamic, and wicked threat to health around the world in which global inequities were stark and unacceptable. In 2004, 39 million people were living with HIV, 4.9 million new infections occurred, and 3.1 million died of HIV (comparable to the approximately 3.1 million COVID-19 deaths by April 16, 2021) in that year, the vast majority of new infections (>95%) and deaths (>95%) occurred in lower and middle-income countries. At the time, lifesaving antiretroviral therapy was available only in high-income countries, where it was expensive, delivered by highly trained specialists, and accompanied by sophisticated monitoring requirements (e.g., quantitative HIV plasma RNA levels and genotypical analysis). Naysayers warned that providing treatment throughout the world to close these major gaps would lead to “antiretroviral anarchy” and widespread drug resistance. To make progress against HIV, and increase equitability around the world, the world faced many of the same problems that we face today. Was the crux of problem access to antiretrovirals or to medications more generally? Should the priority be on treatment or prevention? Was this a public health problem at all or actually one of poverty and extractive economic systems? Three decades later, HIV treatment is widely available around the world, mostly free of charge, delivered by a robust healthcare workforce, based on stable global financing, and supported by the highest levels of global governance. With all the flaws of the response, from the vantage point of the present, it is clear that progress and greater equity in the response to the HIV epidemic have occurred.

In the HIV response, this progress toward a global response was anchored by advocates and activists who successfully put HIV at the center of political agenda and crafted a globally shared commitment to offer treatment to every person living with HIV anywhere in the world in need [3,4]. This shared formulation of the problem (and therefore shared goals) was a critical first step in the global HIV treatment response. Even though the objective to treat all who needed treatment is crystal clear in retrospect, it was one of many competing perspectives. A few advocated a larger scope (i.e., global progress on a wider range of health conditions), while many championed smaller and “more feasible” objectives, (i.e., emphasizing prevention only without treatment). Yet, the goal to treat every person was ultimately adopted by governments, civil society, and the global agencies and therefore carried legitimacy and force. The Joint United Nations Programme on HIV/AIDS (UNAIDS) was established in 1994 through a UN resolution approved by all 193 member states to provide coordinated and aligned actions on HIV at the highest levels. The Global Fund for HIV, TB, and Malaria was created in 2002 to raise, manage, and disburse billions in funding for these priority pandemics. The first Global AIDS Coordinator to oversee the US government’s investment of billions of dollars to address HIV in 2006 reported directly to the US President, elevating the visibility and prioritization of the HIV response where it was most severe.

This shared agenda to treat all in need was accompanied by the emergence of a remarkable visual heuristic that provided a shared roadmap for progress:—HIV treatment cascade—a simple and immediately accessible representation of the fraction of individuals living with HIV who are diagnosed, linked to care, started on treatment, retained, and virally suppressed. While subject to a range of criticisms (e.g., the cascade does not represent time, real journeys are cyclical), the heuristic rapidly became a universal mental model of the implementation needs for success in the global treatment response for HIV. Today, this framing is shared by virtually all health officials, healthcare workers, implementers, policy makers, and even elected officials addressing HIV anywhere in the world [5]. The shared understanding of the public health approach allowed a remarkable fluency (or 95-95-95 in some places) of action and alignment of goals across the public health environment [6,7]. Comparisons of the cascade in different regions has also sharpened the focus on equity when large differences are apparent.

Finally, the HIV response has used the cascade framework to define clear targets and extract commitments from elected leaders to meet those targets, thereby enabling measurable progress. The overarching UNAIDS strategy for HIV treatment, called “90–90–90," refers to targets in this cascade—that 90% of individuals living with HIV are diagnosed, 90% of diagnosed are on treatment, and 90% of those on treatment are suppressed. Today, virtually every country, state, county, and public health jurisdiction in the world is expected to know how much progress is being made toward 90–90–90 in their own jurisdictions. The International Association of Physicians in AIDS Care’s Fast-Track Cities initiative is one of a number of global policy initiatives based explicitly on obtaining commitments from political leaders and elected officials to meet 90–90–90 cascade targets [8]. The initiative has enrolled over 350 cities around the world—with the mayors of London, Paris, and other cosmopolitan cities all vocal advocates of 90–90–90. Because the cascade is reported, progress toward 90–90–90 can be widely assessed, creating some basis for accountability.

Each of these 3 steps have implications for progress against the problem of an equitable global COVID-19 response. At present, the working global targets for distribution of vaccine have been put forth by COVAX, an alliance between GAVI, Coalition for Epidemic Preparedness, and WHO. COVAX seeks to allocate vaccine to cover 20% of the population in all countries. Yet, is a 20% floor targeting success or capitulating to failure? Is its distribution schema based on population fair, or, as proposed by others, should it be based on COVID-19 disease burden [9]? In order to answer these, and many other critical questions of shared global significance, the issue of equity must take center stage in the global policy making conversation at the highest levels. Ottersen and colleagues observed that “conflicts in interests and power asymmetries” between transnational actors on health (e.g., governments, corporations, and civil society) demand institutions to negotiate, articulate, and advocate for collective interests [10]. While the institutional arrangements may take various forms, a high-level UN meeting to establish a common agenda for equity in the global COVID-19 response attended by all 193 member states—as has been done before for HIV, TB, noncommunicable diseases, and antimicrobial resistance—is a minimum step.

Like the HIV cascade, consensus for a more equitable COVID-19 response must be accompanied by meaningful, interpretable, publicly facing, and credible metrics that explicitly depict equity [11]. Many candidate metrics for that exist, but more work needs to be done to identify a consensus-based set. For example, the Lorenz curve, which has long been used in economics, could be used to depict the distribution of vaccine globally; as depicted in Fig 1, the Gini index has changed little over time, indicating sluggish, if any, progress toward equitable vaccine distribution. Other candidate metrics include adaptations of the Gini coefficient and/or the Palma ratio (the ratio of the richest 10% of the countries share of vaccines to the poorest 40%’s share). Measures based on Atkinson index could be promising because they would weight changes among those with less as compared to changes among those with relatively more [12]. Such shared mental models of the roadmap, in turn, can be used to demand concrete commitments from global agencies, national governments, and other actors, including from industry.

**Fig 1:**
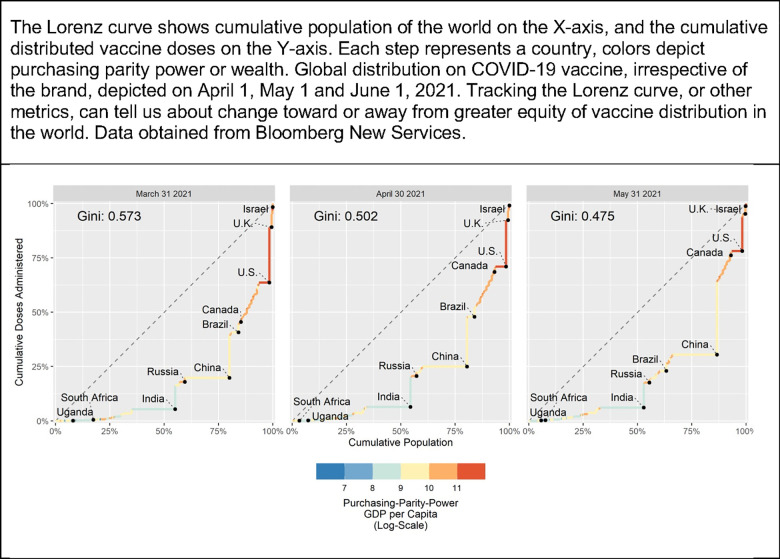
Lorenz curve and Gini coefficient as examples of potential metrics explicitly depicting equity of global distribution of COVID-19 vaccine. COVID-19, Coronavirus Disease 2019; GDP, gross domestic product.

Sometimes, change occurs quickly during windows when the right problems, politics, and policy converge [13]—the COVID-19 pandemic presents a critical threat but also potentially a window to catalyze international collective action that can shape the post-COVID-19 landscape and ensure that global responses to future health threats are more equitable and effective. The barriers to using these steps to achieve greater equity in the global response to COVID-19 are myriad: Not all influential actors globally prioritize equity; agreement on metrics may be elusive; low-quality data behind metrics can mislead; and mechanisms for social accountability often fail [14]. Yet, the alternative is more daunting—the absence of a conversation, metrics, and mechanisms for global equity that is commensurate with its unequivocal place at center of global stage will set a dire path for the future. Not everything in the HIV response has gone well—indeed, opportunities for progress have been missed—but the response demonstrates that wicked problems are not completely intractable, but only if we are committed enough to change them.
